# Holstein Cattle Face Re-Identification Unifying Global and Part Feature Deep Network with Attention Mechanism

**DOI:** 10.3390/ani12081047

**Published:** 2022-04-18

**Authors:** Xiaolang Chen, Tianlong Yang, Kaizhan Mai, Caixing Liu, Juntao Xiong, Yingjie Kuang, Yuefang Gao

**Affiliations:** College of Mathematics and Informatics, South China Agricultural University, Wushan Road, Tianhe District, Guangzhou 510642, China; chenxiaolang@gdrcu.com (X.C.); xfsuo@scau.edu.cn (T.Y.); kz@stu.scau.edu.cn (K.M.); liu@scau.edu.cn (C.L.); xiongjt@scau.edu.cn (J.X.)

**Keywords:** precision dairy farming, cow face re-identification, deep learning, GPN model, GPN-ST model

## Abstract

**Simple Summary:**

Automated and non-intrusive recognition of individual livestock such as a cow or a pig is a vital requirement for obtaining individual information, something which has played a significant role in the intelligent management and welfare of livestock. However, individual cow identification remains a demanding task mainly due to the quite yet subtle inter-class differences in an open herd setting. The objective of this study is to develop a novel unified global and part feature deep network model (GPN) to learn more discriminative and robust features that can facilitate cow face representation for re-identification and verification. The results of various contrast experiments on collecting the large-scale cow face dataset show that the proposed GPN model outperforms the existing representative re-identification methods, and the further improved GPN-ST model has a higher accuracy rate (up by 2.8% and 2.2% respectively) in Rank-1 and mAP, compared with the GPN model. In conclusion, using the proposed framework can effectively ameliorate the performance of cow face re-identification.

**Abstract:**

In precision dairy farming, computer vision-based approaches have been widely employed to monitor the cattle conditions (e.g., the physical, physiology, health and welfare). To this end, the accurate and effective identification of individual cow is a prerequisite. In this paper, a deep learning re-identification network model, Global and Part Network (GPN), is proposed to identify individual cow face. The GPN model, with ResNet50 as backbone network to generate a pooling of feature maps, builds three branch modules (Middle branch, Global branch and Part branch) to learn more discriminative and robust feature representation from the maps. Specifically, the Middle branch and the Global branch separately extract the global features of middle dimension and high dimension from the maps, and the Part branch extracts the local features in the unified block, all of which are integrated to act as the feature representation for cow face re-identification. By performing such strategies, the GPN model not only extracts the discriminative global and local features, but also learns the subtle differences among different cow faces. To further improve the performance of the proposed framework, a Global and Part Network with Spatial Transform (GPN-ST) model is also developed to incorporate an attention mechanism module in the Part branch. Additionally, to test the efficiency of the proposed approach, a large-scale cow face dataset is constructed, which contains 130,000 images with 3000 cows under different conditions (e.g., occlusion, change of viewpoints and illumination, blur, and background clutters). The results of various contrast experiments show that the GPN outperforms the representative re-identification methods, and the improved GPN-ST model has a higher accuracy rate (up by 2.8% and 2.2% respectively) in Rank-1 and mAP, compared with the GPN model. In conclusion, using the Global and Part feature deep network with attention mechanism can effectively ameliorate the efficiency of cow face re-identification.

## 1. Introduction

Automated and non-intrusive recognition and verification of individual livestock such as cows or pigs is a vital requirement for obtaining individual information (e.g., the physical, physiology, health and welfare), something which has played a significant role in controlling the outbreak of critical diseases and monitoring vaccination, traceability, body condition score, activity behavior, and other factors. Various technologies such as tattooing, visual ear tags, radio-frequency identification (RFID) tag and animal biometrics have been used for livestock identification, and extensive literature has been published [1,2,3,4,5,6,7]. With regard to individual cow identification, approaches based on biometrics (e.g., cattle muzzle pattern, facial feature, coat pattern, gait, tailhead and iris pattern) have been widely adopted partly due to their non-invasiveness, efficiency, low cost, and the development of computer vision. To this end, many researchers have utilized representative hand-designed feature descriptors to extract the discriminative features and achieved acceptable identification performance [8,9,10,11,12]. For example, Kumar et al. [10] used the speeded up robust feature and local binary pattern to extract the features at different levels of Gaussian pyramid in the muzzle point image. Moreover, Li et al. [11] utilized Zernike moments to characterize the shape on the region of interest of the cow tailhead image. In Ref. [12], the authors unified gait and texture features acquired from RGB-D images to model the cow appearance. Due to the powerful feature-learning capacity of the deep convolutional neural networks (CNNs) in computer vision, the top-performing approaches have been built upon various CNN models recently [13,14,15,16]. Bhole et al. [17] employed the deep network model to classify 136 classes of Holstein cattle using the infrared and RGB images of the cow’s side and the identification accuracy achieved was 97.54%. In Ref. [18], a R-CNN model was trained for individual identification of Holstein Friesian Cattle. A recent study [19] further used the YOLOv2 and InceptionV3-based long-term recurrent convolutional network, existing CNN-based object detection and recognition models, to respectively detect and identify individual cattle via coat pattern images. In addition, a cow’s feed intake measurement system was proposed in Ref. [20], where the authors used RGB-D camera and deep learning algorithm for cow identification, with the identification accuracy reaching 93.65%. Different from the above individual identification methods for trained individual cows, others have attempted to use multi-view embedding strategy [21] and metric learning algorithm [22] for cow re-identification that identified cattle in previously unseen cattle datasets.

Although encouraging results have been achieved, individual cow identification remains a demanding task mainly due to great intra-class variation, which can be attributed to viewpoint, scale, occlusion, illumination and small inter-class variation caused by details with subtle similarities. Furthermore, most of those methods focus on a fixed set of known cattle, with the same category shared by the training set and the testing set. Consequently, a change in the herd (e.g., additions to the herd) or a transfer to a new herd involves labor-intensive data gathering and labeling, apart from computationally full re-training of a closed-set classifier. In addition, current datasets are relatively small in size, e.g., FriesiaCattle2017 [18] with 940 images of 89 cattle individuals, Holstein cattle dataset [17] with 136 cows, and cattle walking dataset [23] with 528 videos. On these tough issues, little work has been dedicated to the analysis of large-scale cow face re-identification. 

Person re-identification aims to obtain match images of the same identity across different non-overlapping cameras. The key to addressing this challenging task is to learn powerful and discriminative feature representation from the training data. From hand-crafted low-level features used in the early methods [24] to the recent deeply-learned paradigms [25,26,27,28,29], the technology of person re-identification has achieved impressive progress and attained state-of-the-art performance in the public datasets. For example, Yang et al. [30] developed a patch discriminative feature learning network for unsupervised person re-identification by selecting patches from the feature map and learning compact and discriminative features for each patch. Moreover, some research groups [31,32] have combined attention mechanisms with different CNN models to improve feature representation. This was achieved via learning more multi-grained meaningful information from the parts and has achieved positive results in public datasets. Instead of simply learning global or local features, others have attempted to combine them to learn more robust and discriminative feature representation [33]. Furthermore, the unifying in-depth and context-aware learning framework [34] has been proposed for person re-identification that learned not only the features of different parts but also the information relationship between different parts via a hierarchical graph convolutional network. To extract more potential part features, Chen et al. [35] introduced a salience-guided cascaded suppression network to model the feature representation of a pedestrian image by integrating those salient local features with the global features. Due to the advantages of discriminative and robust feature learning from training data and direct utilization to the unseen testing data, it is logical to apply the Re-identification strategies to the exploration of the feasibility of cattle face identification and re-identification. However, apart from the issues shared with person re-identification, researchers who engage in cow re-identification are confronted with many additional challenges. First, different individual cows of the same herd share very similar face appearances, and the distinctions among them are quite subtle and local, creating a tough task to distinguish them from each other. Second, it is more difficult to learn the facial features of cows with identifiable elements and parts (e.g., eyes, ears, and nose) because of some interference factors such as hair on their faces or texture changes. Finally, their facial features may change obviously in a short period because of a relatively short growth cycle, leading to great variances in the same cattle. Due to these challenges, the existing person re-identification approaches cannot meet the large-scale cattle re-identification requirements.

To address the above issues, we propose a novel unified global and part feature deep network (GPN) framework that cooperates with three branch modules, capturing both the global feature and the local detail to enhance the feature representation discriminability. To this end, three branch modules, the Middle branch, the Global branch and the Part branch, are developed based on the feature maps from the backbone network ResNet50 [36]. The Global branch and the Middle branch, adaptively and respectively, learn the high-dimensional and middle-dimensional global feature representation from different convolution layers, and the Part branch captures the subtle differences in different parts of a cow face. Once those features are determined, the fusion of features will also be generated as a final cow face feature representation to measure the similarity of the image pairs such as the images to be recognized and the images in Gallery. Moreover, we further explore the attention mechanism in the Part branch to adaptively select the semantic parts. In this way, the proposed framework can effectively leverage the distinctive context of the complete part region to avoid learning corrupted information. 

The contributions of this study are as follows. (i) We propose a simple but generic GPN framework that adaptively exploits the global information and fine-grained local details based on feature maps of different hidden layers to learn more discriminative feature representation for cow face re-identification. (ii) Attention mechanism extension of the framework is further developed, which allows our method to leverage the most informative parts of a cow face to improve its performance. (iii) We introduce a new large-scale cow face dataset for individual cow re-identification to promote livestock animal identification research.

## 2. Materials and Methods

### 2.1. Construction of Cow Face Dataset

**Figure 1:**
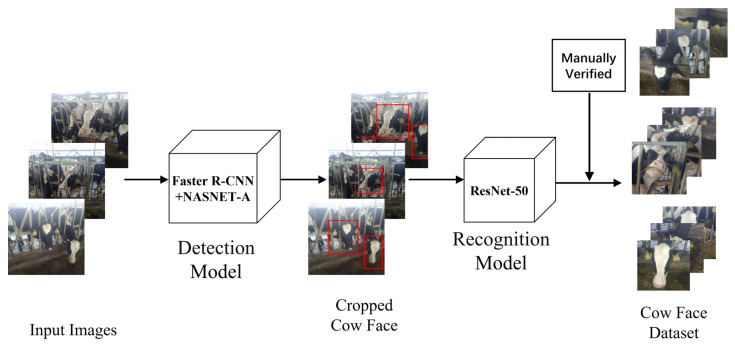
Generation process of cow face dataset.

To verify the efficiency of the proposed model for cow face re-identification, the data of cow images were collected in an open farming environment by using multi-image capture equipment, and a large-scale cow face dataset was constructed, which contained 130,000 images in total with 3000 cows under different conditions (e.g., occlusion, view variance, blur, and illumination variance). To this end, we first obtained the original images of cows (in full length or in half length, possibly with several cows in the same image) captured by mobiles or digital cameras from various viewpoints from a dairy cattle farm in South China. After preliminary data screening and data cleansing, the cow face images can be detected from the raw data based on our improved Faster R-CNN +NASNET-A detection model. Next, we made coarse classification by training the ResNet50 classification model, and manually conducted verification, as shown in Figure 1. Finally, by referring to the data format and data partitioning of the person re-identification dataset (e.g., Market-1501 [37] and CUHK03 [38]), we constructed the training set and the testing set of the cow face dataset, building a dataset which can be used in the follow-up experiments.

The dataset, which contains 130,000 cow face images, is divided into a training set and a testing set. The training set covers 2000 cow face categories, and each category covers 50 cow face images, so there are 100,000 cow face images in total. The testing set covers 1000 cow face categories totally different from those of the training set, which is divided into Query and Gallery respectively with 10 and 20 cow face images of each category, and there are 30,000 cow face images in total. We expect to construct a gallery subset with fewer images and better re-identification performance in actual application. Therefore, in the Gallery each category is further divided, respectively covering only 1, 2, 3, 4, 5, 10, 15 and 20 (representing all images) cow face images in various gallery subsets, in order to verify the performance of the proposed re-identification network from multiple angles.

**Figure 2:**
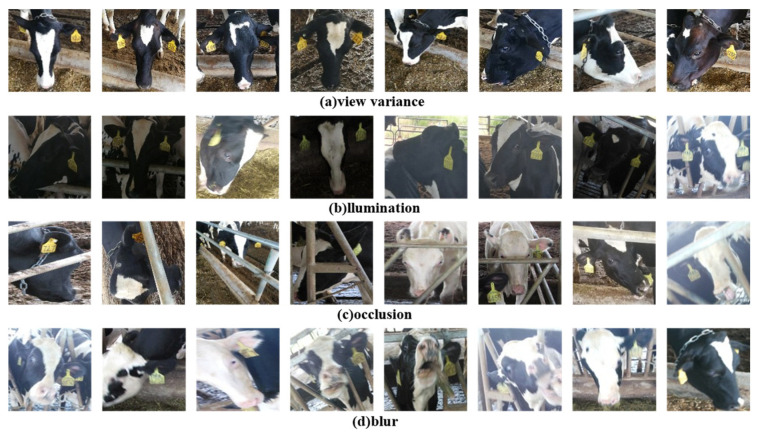
Data distribution of cow face dataset.

**Figure 3:**
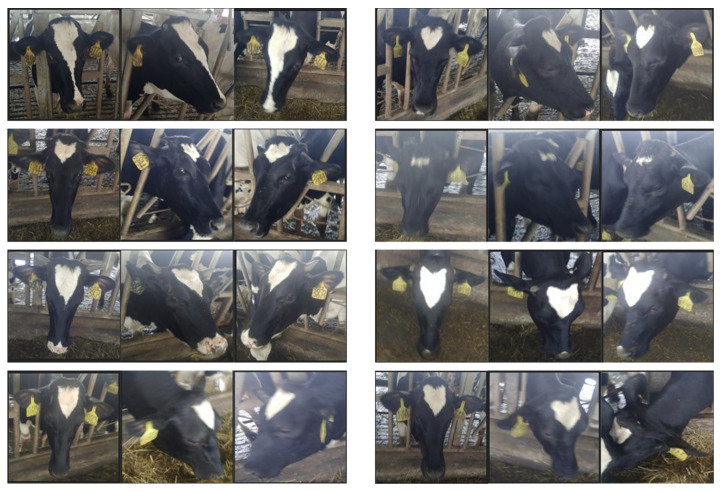
Comparisons of intra-class gap and inter-class gap in dataset. Three cow face images of the same cow are in a row.

In addition, the dataset contains cow face images captured from different angles, including left, full-frontal, and right faces. It also involves cow face data under various conditions, such as illumination, occlusion and blur, which is shown in Figure 2. The above dataset situations are included to ensure the image diversity so that the dataset is both complex and challenging, thus accordingly improving the generalization and robustness of the proposed model. Some samples are selected from the dataset, which is shown in Figure 3. In each group, 3 cow face images are in a row, which are the full-frontal, left and right faces of the same cow. According to the observation, the same cow face shows significant differences from different views. Through vertical comparison, different cows show significant similarities in their facial texture. Therefore, re-identification becomes more challenging.

### 2.2. The Proposed Method

In this section, a detailed description of the proposed GPN framework, which takes advantage of the global and the local features to capture the discriminative and robust information, is presented. Moreover, spatial transform extension of the proposed model (the GPN-ST), which incorporates attention mechanism into the Part branch to further improve the performance of the proposed method, is also developed.

#### 2.2.1. Pipeline Overview 

**Figure 4:**
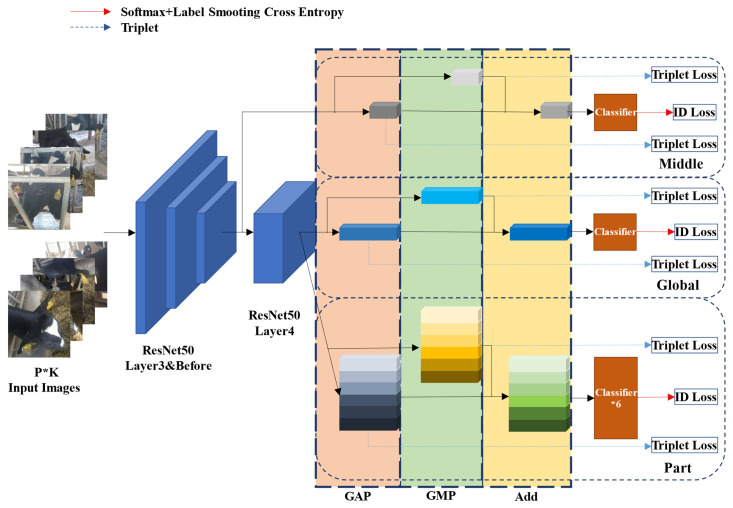
The architecture of the proposed GPN model which cooperates with three branch modules, capturing both the global feature and the local detail to enhance the feature representation discriminability.

**Figure 5:**
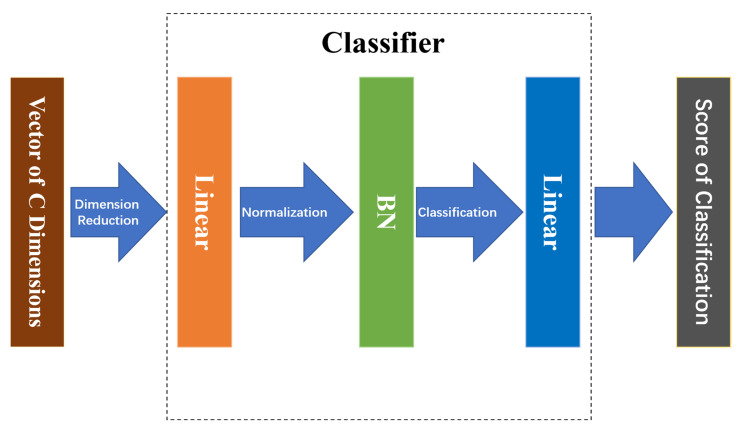
Architecture of Classifier.

The pipeline of the proposed GPN model is shown in Figure 4. First, the model employs ResNet50 as the backbone network, which has removed the final pooling layer and full connecting layer. Second, the convolution step of sub-sampling in Layer4 of the backbone network ResNet50 is adjusted to 1 to enhance feature mapping to improve network performance. Furthermore, the proposed network model contains three branches with different characteristics. The Middle branch and the Global branch separately extract the global features of middle dimension and high dimension from the images through the network, and the Part branch extracts the local features via partition in the unified block, all of which are integrated to act as the feature representation for cow face re-identification. Finally, as is shown in Figure 5, a three-layer structural classifier is used. It contains a linear layer which can reduce the multi-dimensional vector to a vector of 512 dimensions, a Batch Normalize (BN) layer, and a final linear layer for classification.

(1) Global branch

As is shown in Figure 4, the Global branch is in the middle part, which extracts the global features of the high dimension from the images. Similar to the common identification network in feature extraction procedures, this branch processes the feature maps generated from the Layer4 of backbone network ResNet50 first, which also means the feature maps are separately generated through the whole backbone network, Global Average Pooling (GAP) and Global Max Pooling (GMP), and then the feature vector *G_Avg and G_Max* both in 2048 dimensions are respectively generated. Then a new feature vector *G* is obtained by adding *G_Avg* to *G_Max*. Specifically, the feature vector *G* is put into the classifier shown in Figure 5. Then, *G_Down* is generated through the first linear layer and the BN layer. Furthermore, the score of classification prediction is acquired through the final linear layer. In this branch, triplet loss is used to calculate the loss of *G_Avg* and *G_Max* separately, which is shown as follows:

(1) $$L_{t}=\sum_{i}^{N}{\left[D_{ia,ip}^{2}-D_{ia,in}^{2}+\alpha \right]}_{+}$$

Here, $D_{ia,ip}^{2}$ represents the distance of positive ia and ip. $D_{ia,in}^{2}$ represents the distance of negative pairs ia and in. α is a pre-defined distance margin such that the margin of $D_{ia,ip}^{2}$ and $D_{ia,in}^{2}$ is less than α.

The softmax cross entropy loss after label smoothing, which is shown as Formula (2), is employed to calculate the loss of the score of classification prediction.

(2) $$q_{i}=\{\begin{array}{cc} 1-\frac{N-1}{N}\varepsilon , & \mathrm{if}\;i=y\\ \varepsilon /N, & \mathrm{otherwise} \end{array}$$

Here $q_{i}$ is the *i*th element of actual value of classification prediction, y refers to the correct classification category, N is the total number of categories, and ε is a pre-set smoothing parameter. Based on the Formula (2), the result value of true classification can be less than 1, and the result for false classification also has a relatively small value.

(2) Middle branch

As is shown in Figure 4, the Middle branch is in the upper part. This branch can extract the global features of the middle dimension. Inspired by the MiddleNet in Ref. [39], this branch extracts the global features of the middle dimension to enrich the feature information, thus improving the performance of the global features. Similar to the Global branch, the Middle branch extracts feature maps from the Layer 3 of backbone network ResNet50, and then separately processes the extracted feature maps through Global Average Pooling (GAP) and Global Max Pooling (GMP) to respectively generate two feature vectors (*M_Avg* and *M_Max*) both in 1024 dimensions, and a new feature vector *M* in 1024 dimensions is obtained by adding *M_Avg* to *M_Max*. Then the feature vector *M* is put into the corresponding classifier, generating vector *M_Down* in 512 dimensions through the first linear layer and the BN layer, acquiring the score of classification prediction through the final classification linear layer. Similarly, the loss of *M_Avg* and *M_Max* is calculated by using triplet loss (Equation (1)), and the loss of the score of classification prediction is calculated through softmax cross entropy loss after label smoothing (Equation (2)).

(3) Part branch

As is shown in Figure 4, the Part branch is in the lower part. By separating local parts and learning to turn the parts information into corresponding feature descriptions, this branch can extract the local feature information which is likely to be ignored or narrowed in the global features, so that the feature information of associated feature representation used as the final comparison basis can be increased and its representation can be improved. Specifically, the Part branch processes the feature maps generated through the whole backbone network ResNet50 by using GAP and GMP to respectively construct two new feature maps. Then these two maps can be expanded to obtain two feature vectors (*P_Avg* and *P_Max*) both in 12,288 dimensions, and the feature map *P* in new size can be acquired by overlapping these two feature vectors. On this basis, by obtaining 6 horizontal/vertical blocks with identical sizes via even partition of the feature map *P*, 6 local feature vectors *P_Down* in 2048 dimensions are extracted. The 6 local feature vectors are then input respectively in 6 classifiers. Each feature vector through the first linear layer and the BN layer of the corresponding classifier generates vector in 512 dimensions, and 6 scores of classification prediction are acquired through the final linear layer, with all these scores corresponding to the 6 blocks. Similarly, the loss of *P_Avg* and *P_Max* is calculated by using triplet loss (Formula (1)), and the loss of 6 score of classification prediction is calculated through softmax cross entropy loss after label smoothing (Formula (2)).

#### 2.2.2. Extension with Attention Module

**Figure 6:**
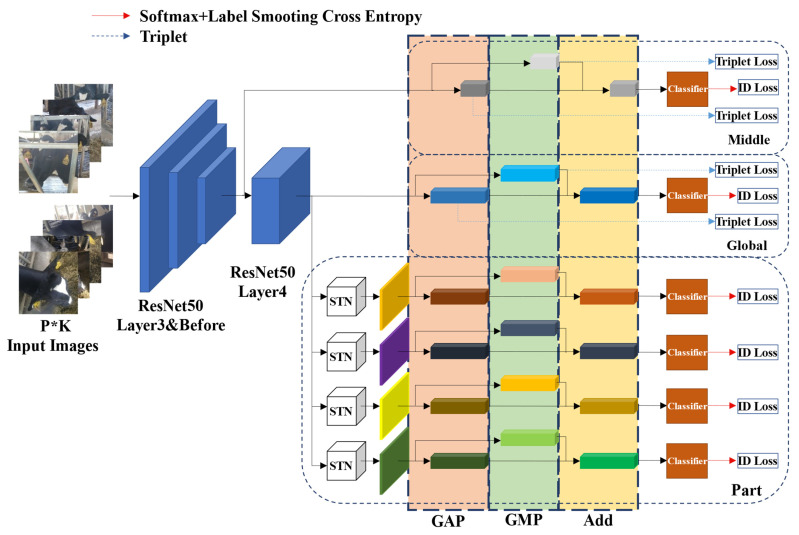
Architecture of the proposed GPN-ST model. The GPN-ST model is improved in the Part branch used to extract the local regions via four STN modules.

In the GPN model, a local subdivision method used in the Part branch, which can obtain blocks with identical size via even partition, is simple and effective, and achieves good results in the experimental testing. However, the existing problems are inadequate alignment among the blocks from different images and insufficient split of regions with complete local information. Therefore, based on the GPN model, the Part branch is further expanded with the incorporation of an attention mechanism, adopting the Spatial Transformer Network (STN) module to replace the strategy of the local region extraction via even partition for better feature representation. The Extension with the attention module is the GPN-ST model, and its network architecture is shown in Figure 6. Identical to the GPN model in the backbone network, the Global branch and the Middle branch, the GPN-ST model is improved in the Part branch used to extract the local regions via four STN modules so that more distinctive and subtle information of the cow face can be acquired. For this end, first, the STN modules are used to extract the regions with distinctive information from the feature maps generated from the whole backbone network ResNet50, thus obtaining 4 new feature maps. Next, each new feature map is used to generate 2 vectors in 2048 dimensions through GAP and GMP procession, and a new vector in 2048 dimensions is also generated by overlapping the 2 vectors. Finally, each vector (all in 2048 dimensions) generated by the STN modules is fed in its corresponding classifier, and the feature vector in 512 dimensions can be generated through the first linear layer and the BN layer, so the score of classification prediction is acquired through the final linear layer.

In training, the loss of the score of classification prediction from the four STN modules in the Part branch is calculated by the softmax cross entropy loss after label smoothing (Formula (2)). In testing, similar to the GPN model, six feature vectors (all in 512 dimensions) from the three branches (Global, Middle, and Part) are stitched to acquire the vector in 3072 dimensions, which is used as the final feature representation for comparison.

For the STN modules, a 2D affine transformation matrix (Formula (3)) is used to restrict the transformation parameter matrix generated by the network. sx and sy are scaling parameters, representing respectively the ratio of length and width of the transformed region on the original images and the length and width of the original images. In contrast, tx and ty are translation parameters, representing respectively the coordinate position of the central point in the original images after the transformation. The matrix is only used to transform the scale and the shift to ensure that the spatial information between the extraction region and the original image are consistent.

(3) $$[\begin{array}{lll} sx & 0 & tx\\ 0 & sy & ty \end{array}]$$

To further restrict the other parameters in Equation (3), correction and constraint in the form of loss are used during the parameter learning. First, the ranges of sx and sy are restricted via the following Equation (4):

(4) $$L_{ss}={\left(max\left(\left|sx\right|-\alpha ,0\right)\right)}^{2}+{\left(max\left(\left|sy\right|-\alpha ,0\right)\right)}^{2}$$

where α is a constraint parameter determined as 0.5 in the experiment. That is, the values of sx and sy are restricted in the range of [−0.5, 0.5]. Second, the positive constraint on sx and sy is bounded as shown in Equation (5) below:

(5) $$L_{SP}=max\left(0,\beta -sx\right)+max\left(0,\beta -sy\right)$$

where β is a constraint parameter determined as 0.1 in the experiment. That is, by restricting the value, sx and sy cannot be less than 0.1, and the two parameters are restricted to be positive values only so as to prevent be mirrored after the scaling transformation of the images. Third, the range of tx and ty is also restricted as shown in Equation (6) below:

(6) $$L_{TS}={\left(max\left(\left|tx\right|-\gamma ,0\right)\right)}^{2}+{\left(max\left(\left|ty\right|-\gamma ,0\right)\right)}^{2}$$

where γ is a constraint parameter determined as $\sqrt{2}/2\approx 0.71$ in the experiment. If the part of the extracted region is beyond the original image, the subsequent identification will be affected. Therefore, restricting the central point can make this phenomenon less likely to happen after the translation in a certain range of coordinate system in the original image. Furthermore, we set up the mutual exclusion constraint on tx and ty is defined, as shown in Equation (7) below:

(7) $$L_{TE}=\sum_{i}^{N}\sum_{j>i}^{N}\left(max\left(0,\delta -\left({\left(tx_{i}-tx_{j}\right)}^{2}+{\left(ty_{i}-ty_{j}\right)}^{2}\right)\right)\right)$$

where δ is a constraint parameter which is determined as 0.4 in the experiment. N is referred to as the number of the STN module used in the Part branch. Equation (7) is used to restrict the squared value of distance between the central points extracted by each STN module, which should not be less than δ, so that there is a mutual exclusion constraint between each extracted region. Therefore, different parts of the image can be extracted by each STN module. Finally, all the losses mentioned above are combined to acquire the comprehensive constraint loss of parameter matrix generated by the STN modules, which is shown in Equation (8) below:

(8) $$L_{STN}=L_{SS}+\lambda_{1}L_{SP}+L_{TS}+\lambda_{2}L_{TE}$$

where $\lambda_{1}$ and $\lambda_{2}$ are both determined as 0.1 in the experiment.

### 2.3. The GPN and the GPN-ST Model Training

During the GPN model training, we use the joint loss caused by the three branches mentioned above to optimize the network parameters. Specifically, we add up two triplet losses using Equation (1) in each branch, which means 6 losses in total, to get the total triplet loss $L_{T}$, and get the total classification loss $L_{ID}$ by adding up each classification loss using Equation (2) in three branches (with one in the Global branch, one in the Middle branch, and six in the Part branch). The function of final total loss of the GPN network is as follows:

(9) $$L_{GPN}=L_{T}/6+L_{ID}/8$$

where 6 is referred to as 3 branches with 6 triplet loss in total; similarly, 8 is referred to as 8 classification loss in total. During the testing, one *G_Down* from the Global branch, one *M_Down* from the Middle branch and six *P_Down* from the Part branch, which are all in 512 dimensions and eight vectors in total, are jointed to obtain a vector in 4096 dimensions, which will be used in the testing as the final feature representation for similarity comparison.

For the GPN-ST model, the overall loss is shown in Equation (10) below:

(10) $$L_{GPN-ST}=L_{TL}/4+L_{CL}/6+L_{STN}$$

where $L_{TL}$ and $L_{CL}$ are total triplet loss using Equation (1) and total classification loss using Equation (2) respectively. Since the triplet loss is not used in the improved Part branch, the divisor of average triplet loss is determined as 4. In the same way, only 4 STN modules are employed in the Part branch so that the divisor of average classification loss is determined as 6 (with one in the Global branch, one in the Middle branch, and four in the Part branch).

### 2.4. Experimental Setup

The experiment is conducted using an apparatus with an NVIDIA GTX 1080Ti graphics card in the Ubuntu 18.04LTS system. The experimental environment framework is based on Python 3.7 and PyTorch 1.0.

For the network parameters, the backbone network ResNet50 of our proposed GPN model and GPN-ST model uses initialized parameters of a pre-training model on ImageNet. For the network input, we set the size of the images as 384×192. In training, batchsize is set as 32, with random flip horizontally and random erasure used. Due to optimization using the triplet loss, a random identify sampler is set up to randomly select 8 categories in each batch and 4 samples in each category. During the training, SGD with its use weight attenuation set as 0.0005 and momentum set as 0.9 is used as optimizer, and the initial learning rate is set as 0.02 and divided by 10 descent when the epoch is 90 and 120. During the testing, batchsize is set as 8.

During the validation process of the re-identification test, to set the similarity degree or distance is required as a basis of comparison between the images to be recognized and the images in the Gallery. In the present study, cosine similarity is used for this purpose, and its formula is as follows:

(11) $$\cos\left(\theta \right)=\frac{A\cdot B}{||A||\times ||B||}$$

where *A* and *B* are two vectors for comparison; · means using the vector dot product, and × means using normal multiplication. Larger cosine value represents smaller angle and more similarities in the 2 vectors. Conversely, more differences can be found between the 2 vectors. 

In terms of performance evaluation, we select the accuracy of Rank-1, Rank-5 and Rank-10, as well as the mean average precision (mAP), which are commonly used in recognition tasks as the evaluation indicators. Rank-1, Rank-5 and Rank-10 represent, after similarity comparison and ranking, the respective ratio of the numbers of times that there are images in the same category to be recognized and the total numbers of images to be recognized in the top1, top5, and top10 images.

Since the re-identification task is a retrieval ranking problem, we expect to retrieve all images of correct categories while making the images of correct categories in the ranking order, and the mAP is the indicator to evaluate the capacity of the network model. mAP is the average value of the retrieval precision (*AP*) of all images to be recognized, and the *AP* of each image can be obtained using the Equation (12) below:

(12) $$AP=\frac{\sum_{i}^{N}i/p_{i}}{N}$$

where *N* represents the number of images in the gallery in the same category to be recognized, and i represents the image that occurs for the ith time in a correct category, and $p_{i}$ represents the order of the image that occurs for the ith time in this ranking.

In addition, the testing set is divided into Query and Gallery, while Gallery contains subsets covering 1, 2, 3, 4, 5, 10, 15 and 20 images in each category respectively. For convenience, these subsets in Gallery are represented as Gallery 1, Gallery 2, …, Gallery 15, Gallery 20, etc. 

## 3. Results

### 3.1. Comparisons with the Existing Methods

To validate the re-identification performance of the models proposed in this study, we made comparisons and analyses among our proposed network models (the GPN and the GPN-ST), mainstream baseline models (MiddleNet [39], ResNet50 [36]), and the person re-identification approaches (PCB [40], MGN [41]). The ResNet50 model is a classical identification model, so it acts as a baseline of identification performance, while the PCB and the MGN are the existing representative person re-identification models. The recognition results in Gallery1 are shown in Table 1. It can be found that the indexes of Rank and mAP in our proposed models were all superior to those in the mainstream methods. In Rank-1 accuracy and mAP, the performance of the GPN model was 87.2% and 89.1% respectively, up by 6.3% and 5.8% respectively compared with the ResNet50 used as baseline, up by 4.4% and 3.6% respectively compared with the MiddleNet, up by 1.9% and 1.8% respectively compared with the PCB, up by 0.3% in Rank-1 accuracy but down by 1.6% in mAP compared with the MGN. As for the GPN-ST model, the performance was 90.0% in Rank-1 accuracy and 91.3% in mAP, up by 2.8% and 2.2% respectively compared with the GPN to be improved, up by 9.1% and 8.0% respectively compared with the ResNet50 used as baseline, up by 7.2% and 5.8% respectively compared with the MiddleNet, and up by 4.7% and 4.0% respectively compared with the PCB. Unlike the GPN model, in Rank-1 accuracy and mAP, the GPN-ST outperformed the MGN, up by 3.2% and0.5% respectively. Accordingly, in accuracy of Rank-5 and Rank-10, the methods proposed by us also achieved reliable identification performance.

Moreover, tests on Gallery 2, Gallery 3, Gallery 4, Gallery 5, Gallery 10, Gallery 15 and Gallery 20 were conducted to validate the recognition performance of our proposed models in different gallery sets with different numbers of images. The Rank-1 accuracy and mAP are shown in Table 2 and Table 3 respectively. According to Table 2, our proposed GPN and GPN-ST models both outperformed the existing mainstream recognition models, with their average recognition rates being 95.88% and 94.75%, up by 3.63% and 2.5% respectively compared with ResNet50 used as baseline, while the average recognition rate of the GPN-ST model was up by 2.9%, 1.79% and 1.45% respectively compared with the MiddleNet, PCB and MGN models. The better performance of the proposed models can be attributed to the fact that they can capture not only the global features but also the local details of cow face by using different branches, thus making a more discriminative representation of the cow face details by applying data fusion in the areas where cow face textures are similar.

In addition, with more data in the gallery set, the recognition performance of the models proposed by us also gradually became better, and a preferable balance could be achieved between the recognition performance in each model and the number of images in the gallery set when the numbers of images were increased to 4 and 5. In terms of mAP, our proposed GPN-ST model performed the best in most gallery sets, although compared with the MGN model the variation amplitude was 0.1% in the gallery sets with 10, 15, and 20 images, as shown in Table 3. Our proposed GPN model outperformed the other models except for the MGN model because the re-ranking algorithm was used in the MGN model in the test.

### 3.2. Comparisons of Each Module of the GPN Model

The satisfactory recognition performance of the proposed GPN model can be attributed to its integration of local features and global features of middle dimension and high dimension, as well as its combination of GAP and GMP. Meanwhile, classification loss and triplet loss are simultaneously used to optimize the network. This conclusion results from the contrastive analysis of the GPN model simplification experiment (Table 4) in which the GPN model will degenerate into ResNet50 model if the Global branch is considered only (Simple Global), while the GPN model will degenerate into the PCB model if the Part branch is considered only (Simple Part).

According to Table 4, if a network model with GAP was used only (the row corresponding to Only GAP in Table 4), the Rank-1 accuracy and mAP was 84.4% and 86.6% respectively, down by 2.8% and 2.5% compared with the complete GPN model (the row corresponding to GPN(Ours) in Table 4); if a network model with GMP was used only (the row corresponding to Only GMP in Table 4), the Rank-1 accuracy and mAP was 83.8% and 86.0% respectively, down by 3.4% and 3.1% compared with the complete GPN model. The performance degradation mentioned above can be attributed to the fact that pooling treatment is a down-sampling practice, which results in partial loss of feature information despite data compression and dimensionality reduction. By contrast, the GPN model simultaneously uses the above-mentioned two pooling treatments and superposes the results. Compared with a single pooling practice, the GPN model can, to a certain extent, reduce the loss of feature information and enhance the feature representation, thus further improving the recognition performance.

Moreover, under the prerequisite of using the above-mentioned two pooling strategies, the recognition performance of a multi-branch model is superior to that of a single branch model. As shown in Table 4, the Rank-1 accuracy and mAP of the network with the Global branch and the Part branch were 86.1% and 88.1% respectively, up by 5.2% and 0.8% respectively in Rank-1, and up by 4.8% and 0.8% respectively in mAP, compared with the network with the Global branch only or the Part branch only. However, the performance was down by 1.1% and 1.0% respectively, compared with the complete GPN model, which indicates that the global features of middle dimension in the middle layer can provide more information to further enrich the global feature representation.

### 3.3. Comparisons between the GPN and the GPN-ST Models

**Figure 7:**
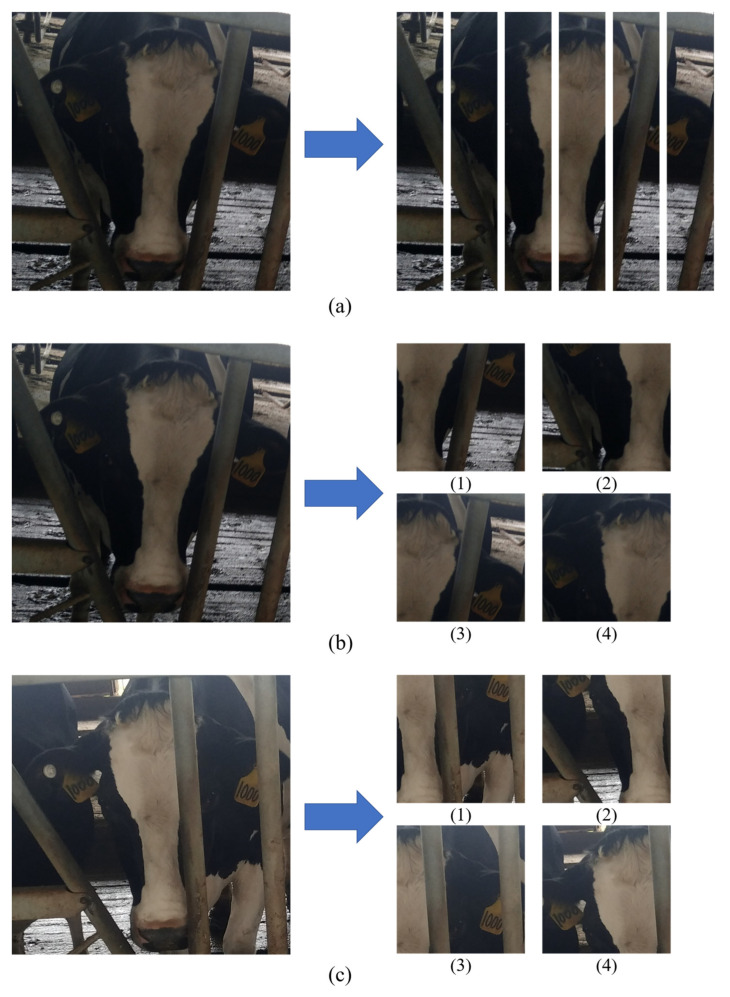
The extracted region visualization of the GPN and GPN-ST models. The left and right column of (**a**) separately represents the input image and the even partitions. The left and right columns of (**b**,**c**) separately represent the input images and the extracted the local regions with STN module.

In the GPN model, the Part branch uses a simple local dividing method which gets unified blocks via even partition achieving positive results. However, there are still shortcomings in this method. For instance, some blocks are not lined up exactly, and some regions with complete global information might be divided (the right column of (a) in Figure 7). By comparison, the GPN-ST model keeps the strengths of the GPN model, for its Part branch has been improved to further optimize feature representation. The visual effect of its extracted regions is shown in Figure 7. According to Figure 7, from the input cow face images (the left columns of (b) and (c) in Figure 7), four STN modules in the GPN-ST model successively extract the local regions of left face, right face, left ear and forehead, as well as right ear and forehead, all of which have provided adequate and discriminative information for the subsequent identification task. In addition, it can be seen from Figure 7b,c that the STN module could basically extract complete local regions in the images with full-frontal faces and incomplete side faces. Furthermore, the regions extracted from the same STN module are one-to-one corresponding between images of the same groups. Compared with the method of obtaining unified blocks via even partition (Figure 7a), this strategy can solve the problem of the local regions alignment and ensure regional information completeness. According to the experimental results in Table 1, Table 2 and Table 3, the amelioration is obvious for this strategy has improved the performance of cow face re-identification. Meanwhile, no extra supervision information is required in training using the method. Therefore, the recognition performance is further improved with the better Part branch in the GPN-ST model.

**Figure 8:**
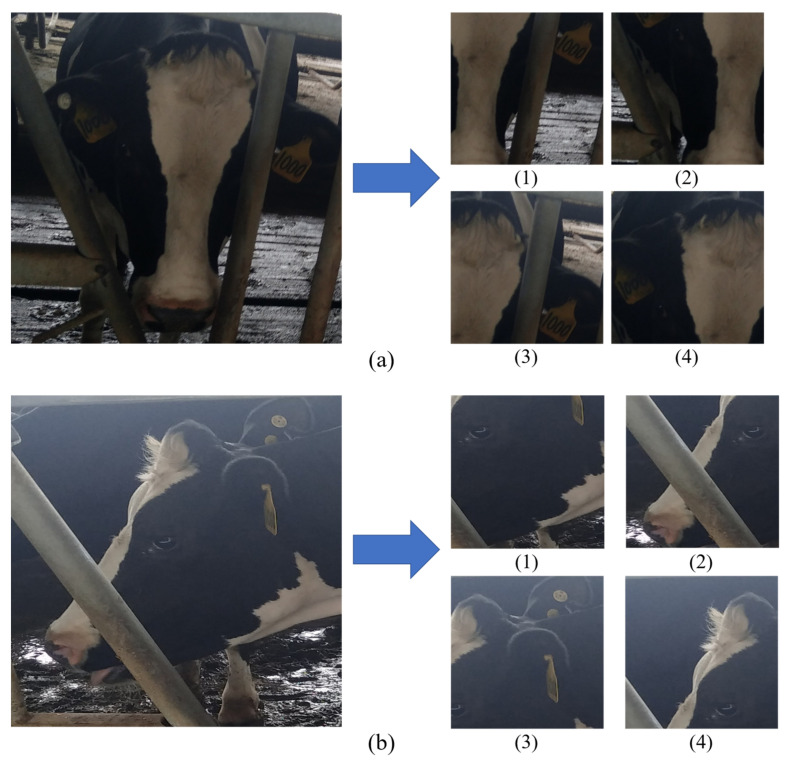
The visualization of failures in the extracted regions of the GPN-ST model. The left columns of (**a**,**b**) are the same cow with different angles. The right columns of (**a**,**b**) separately represent the extracted the local regions with STN module.

However, there are some deficiencies in the GPN-ST model, which are shown in Figure 8 where the visualization of failures in the extracted regions can be found. In Figure 8, the cow face images (the same cow in Figure 7b) are displayed from a different angle. The left column of (a) in Figure 8 is the image of full-frontal face which is the same image of Figure 7a, while the left column of (b) in Figure 8 is the image of side face. It can be found in Figure 8b that, in this case, incomplete and insufficient alignment problems exist in the extracted local regions from the same STN module. The extracted local regions are not complete in the images with complete side face and one-to-one correspondence is hard to realize. The integration of (1) and (2) in Figure 8b is equivalent to the image of left face in (1) of Figure 8a, while the integration of (3) and (4) in Figure 8b is equivalent to the image of left ear and forehead in (3) of Figure 8a. The reason lies in the fact that the four STN modules set in the GPN-ST model can find regions with remarkable local features in the images with full-frontal faces and incomplete side faces, such as the regions with left and right ears, and left and right eyes, yet full occlusion can impede the capture of information in the images with complete side faces. Hence the model segments some regions with local features to reach the required number of modules. Consequently, the completeness of feature regions will be affected, and the problem of alignment will also be caused.

## 4. Conclusions

In this paper, a deep learning GPN network is proposed for cow face re-identification. The network model, with integrated global and local features in high dimension, enhances the discriminatory power of cow face feature representation by extracting features in the middle dimension. The network is optimized by using classification loss and triplet loss, and the recognition performance of the network is improved by using label smoothing on classification loss. Furthermore, based on the GPN model, the GPN-ST model is constructed by integrating the STN module with an attention mechanism in order to extract local regions. The improved model has achieved preferable re-identification performance in a large-scale cow face dataset.

A key-point mechanism can be introduced in the follow-up research to extract the regions of local features so as to improve the accuracy of one-to-one correspondence in the extracted regions. Further, more cow face images with complete side face can be added in the dataset for more adequate data in model training. What is more, the size of the Part branch can be increased when the re-identification performance of the GPN-ST model has been improved. In the future, a balance among the volume of the model, calculating speed and re-identification accuracy can be expected by reasonably compressing and pruning the network model.

## Figures and Tables

**Table 1:** Recognition results of different models in Gallery 1.

Model	Rank-1	Rank-5	Rank-10	mAP
GPN-ST (Ours)	90.0%	95.9%	97.4%	91.3%
GPN (Ours)	87.2%	95.6%	97.4%	89.1%
ResNet50	80.9%	92.2%	94.7%	83.3%
MiddleNet	82.8%	93.8%	95.6%	85.5%
PCB	85.3%	94.4%	96.2%	87.3%
MGN	86.9%	95.5%	97.4%	90.8%

**Table 2:** Rank-1 accuracy of different models in different gallery sets (%).

Model	Gallery 1	Gallery 2	Gallery 3	Gallery 4	Gallery 5	Gallery 10	Gallery 15	Gallery 20
GPN-ST (Ours)	90.0%	94.9%	95.9%	96.5%	96.6%	97.4%	97.7%	98.0%
GPN (Ours)	87.2%	92.6%	94.7%	95.6%	95.7%	97.1%	97.4%	97.7%
ResNet50	80.9%	89.5%	92.0%	92.9%	93.7%	95.7%	96.5%	96.8%
MiddleNet	82.8%	90.2%	92.8%	93.9%	94.3%	96.1%	96.7%	97.0%
PCB	85.3%	91.8%	94.0%	94.8%	95.4%	96.5%	97.4%	97.5%
MGN	86.9%	92.1%	94.2%	95.1%	95.5%	96.7%	97.4%	97.5%

**Table 3:** mAP of different models in different gallery sets (%).

Model	Gallery 1	Gallery 2	Gallery 3	Gallery 4	Gallery 5	Gallery 10	Gallery 15	Gallery 20
GPN-ST (Ours)	91.3%	91.5%	91.3%	91.4%	91.1%	91.2%	91.2%	91.0%
GPN (Ours)	89.1%	88.6%	88.7%	88.7%	88.3%	88.3%	88.4%	88.3%
ResNet50	83.3%	83.6%	83.0%	83.0%	82.5%	82.6%	82.7%	82.6%
MiddleNet	85.5%	85.8%	85.1%	85.6%	84.8%	84.8%	85.0%	84.8%
PCB	87.3%	87.3%	87.3%	87.3%	87.0%	87.0%	87.1%	87.0%
MGN	90.8%	90.2%	90.3%	90.8%	90.7%	91.3%	91.8%	91.5%

**Table 4:** Results of different module combinations of the GPN model.

Model	Rank-1	Rank-5	Rank-10	mAP
GPN (Ours)	87.2%	95.6%	97.4%	89.1%
Only GAP	84.4%	94.0%	96.4%	86.6%
Only GMP	83.8%	94.0%	96.4%	86.0%
Simple Global	80.9%	92.2%	94.7%	83.3%
Simple Part	85.3%	94.4%	96.2%	87.3%
Global + Part	86.1%	94.8%	96.4%	88.1%
Global + Middle	86.2%	94.4%	96.4%	88.1%
Part + Middle	85.7%	94.9%	96.9%	87.7%

## Data Availability

Data sharing not applicable.
